# Facile biosynthesis of Ag–ZnO nanocomposites using *Launaea cornuta* leaf extract and their antimicrobial activity

**DOI:** 10.1186/s11671-023-03925-2

**Published:** 2023-11-17

**Authors:** Elizabeth Makauki, Stanslaus George Mtavangu, Onita D. Basu, Mwemezi Rwiza, Revocatus Machunda

**Affiliations:** 1 School of Materials Energy Water and Environmental Sciences, Nelson Mandela African Institution of Science and Technology, Arusha, Tanzania; 2 Department of Chemical Engineering, Faculty of Engineering Sciences, KU Leuven, Leuven, Belgium; 3 Department of Civil and Environmental Engineering, Faculty of Engineering and Design, Carleton University, Ottawa, Canada; 4 Department of Chemistry, Dar es Salaam University College of Education, Dar es Salaam, Tanzania

**Keywords:** Green synthesis, *Launaea cornuta*, Metabolites, Nanoparticles, Microbial growth inhibition

## Abstract

The quest to synthesize safe, non-hazardous Ag–ZnO nanoomposites (NCs) with improved physical and chemical properties has necessitated green synthesis approaches. In this research, *Launaea cornuta* leaf extract was proposed for the green synthesis of Ag–ZnO NCs, wherein the leaf extract was used as a reducing and capping agent. The antibacterial activity of the prepared nanoomposites was investigated against *Escherichia coli* and *Staphylococcus aureus* through the disc diffusion method. The influence of the synthesis temperature, pH, and precursor concentration on the synthesis of the Ag–ZnO NCs and antimicrobial efficacy were investigated. The nanoparticles were characterized by ATR-FTIR, XRD, UV–Vis, FESEM, and TEM. The FTIR results indicated the presence of secondary metabolites in *Launaea cornuta* which assisted the green synthesis of the nanoparticles. The XRD results confirmed the successful synthesis of crystalline Ag–ZnO NCs with an average particle size of 21.51 nm. The SEM and TEM images indicated the synthesized nanoparticles to be spherical in shape. The optimum synthesis conditions for Ag–ZnO NCs were at 70 °C, pH of 7, and 8% silver. Antibacterial activity results show Ag–ZnO NCs to have higher microbial inhibition on *E. coli* than on *S. aureu*s with the zones of inhibition of 21 ± 1.08 and 19.67 ± 0.47 mm, respectively. Therefore, the results suggest that *Launaea cornuta* leaf extract can be used for the synthesis of Ag–ZnO NCs.

**Supplementary Information:**

The online version contains supplementary material available at 10.1186/s11671-023-03925-2.

## Introduction

During the last decade research in metal and metal oxide nanomaterials has gained popularity due to their distinctive properties and potential applications in various environmental and health fields. Metal and metal oxide nanomaterials are characterized by their small size (1–100 nm) and shape which results in a higher surface area-to-volume ratio influencing and improving the optical, catalytic, electrical, magnetic, and conductive properties of these materials [1–4]. Silver nanoparticles (Ag NPs), in particular, stand out amongst metal nanoparticles and their bulk counterparts due to their remarkable features and high activity potential which has led to significant advancements in several fields including pharmaceuticals, agriculture, biosensors, and water treatment [5, 6]. These nanoparticles exhibit exceptional resistance against microorganisms, making them potent biocidal agents against both gram-positive and gram-negative bacteria. Notably, Ag NPs possess high thermal stability, low volatility and toxicity towards mammalian cells making them preferred in health-related applications [7]. Leveraging their antimicrobial properties, Ag NPs are extensively used in controlled and targeted drug delivery systems, capitalizing on their low toxicity and biocompatibility [8]. Furthermore, their high surface plasmon resonance makes them ideal candidates for the development of biosensors, showcasing their versatility and potential for scientific and technological innovations [3, 9–11]. Despite their great capacity for microbial growth inhibition, the reactive nature of Ag NPs associated with high inter-particle attraction and surface forces consequently results in agglomeration which leads to a reduction of the Ag NPs antimicrobial properties [12–14]. To prevent agglomeration and thereby maintain effective microbial inhibition, Ag NPs may be doped into semiconductor-based heterostructures, such as ZnO therefore making the nanoparticles multifunctional [11, 15–17]. The agglomeration and particle size are controlled as the Ag NPs are attached in the ZnO structure, therefore, reducing self-inter-particle attraction [13]. Shreema et al. [18] observed decreased agglomeration and complete separation of particles on the doping of pure ZnO.

Zinc oxide (ZnO) is a metal oxide semiconductor with a direct wide band gap (3.37 eV). It is an attractive photocatalyst owing to its high photosensitivity, high thermal stability and environmental sustainability [19–22]. Furthermore, ZnO nanoparticles exhibit significant antibacterial efficacy against both gram-positive and gram-negative bacteria even in the absence of light, highlighting their immense potential in combating microbial infections [23]. Doping of metallic elements into ZnO is reported to improve its antimicrobial efficiency as it increases the electron–hole (e–h) separation efficiency therefore enhances photocatalytic activity [16, 24]. Silver-doped zinc oxide nanocomposites, (Ag–ZnO NCs) have been reported to be more effective against gram-positive and gram-negative bacteria, compared to pure ZnO [13, 25]. The association of Ag and ZnO nanoparticles increases the formation of reactive oxygen species (ROS) leading to increased antimicrobial activity [26].

In addition, the agglomeration of silver nanoparticles can be controlled through the use of chemical stabilizing agents such as polyvinylpyrrolidone (PVP), polyvinylalcohol (PVA), hyperbranchedpolyurethane (HP), and polyacrylonitrile (PAN) [27, 28]. Chemical methods include flow—injection, co-precipitation, sol–gel synthesis, micro-emulsion, and hydrothermal reactions [29, 30]. However chemically synthesized nanoparticles have been reported to release toxins at nanoscale and have been linked with elevated causes of diseases such as cancer [5, 31–33]. Chemically synthesized nanoparticles, therefore, may threaten environmental sustainability and limits the human consumption-related applications of these noble materials. Furthermore, physical methods to control agglomeration involve milling, thermal ablation, and grinding but have reported extremely low yields compared to the energy applied [34, 35].

Due to these challenges, there is a need to incorporate the “green chemistry,” technology, an idea that encourages the replacement of conventional chemicals with the non-toxic, and environmentally friendly reducing agents [36–40]. Green synthesis of nanoparticles includes the use of active ingredients from bacteria, fungi, and plants as reducing and stabilizing agents [19, 41, 42]. Plant extracts have been used and greatly influenced the properties of the synthesized nanoparticles depending on the type and quantity of bioactive materials present in the specific plant [43–45]. Plant extracts are deemed to be beneficial than conventional chemicals taking into consideration of their easy accessibility, biodegradability and minimal harmful effects [46]. Yet, limited plant species have had their efficacy in green synthesis validated scientifically. Plant extracts have secondary metabolites such as flavonoids, alkaloids, tannins, proteins, polyphenols, terpenoids, and saponins [1, 14, 47, 48]. Alkaloids, tannins, proteins, polyphenols, and terpenoids act as hydrolyzing/reducing agents reducing metal ions into atoms [28, 49, 50]. The plant's flavonoids and saponins act as capping/stabilizing agents, controlling the size and agglomeration of the nanoparticles during synthesis [51, 52]. As a result, green synthesis becomes a basis for the effective evaluation and development of non-toxic nanomaterials used in drug formulations, water treatment and environmental remediation with limited side effects[35].

As far as we are aware, there is no study that has documented the use of *Launaea cornuta* leaf extract for synthesizing Ag-ZnO NCs. This research therefore explores the novel potential of *Launaea cornuta* leaf extract in green synthesis of Ag-ZnO NCs from nitrates of Ag and Zinc. *Launaea cornuta* belongs to the wild lettuce family; it is used as a vaso-relaxant, sedative, cough suppressant, expectorant and antiseptic [53, 54]. The root is used in the treatment of gonorrhoea, syphilis, sore throat, cough, and eye infections [55]. Traditionally, *Launaea cornuta* has been used to alleviate inflammatory conditions such as joint pains, earaches and swollen testicles [56]. This plant is known to contain significant amounts of ascorbic acid (vitamin C), phenols, tannins, alkaloids, and flavonoids compounds essential in green synthesis as reducing and capping agents [56–58]. The study further investigates the influence of synthesis temperature, pH of media, and silver dopant concentrations on the synthesized NPs. The antimicrobial efficiency of the synthesized NPs is tested against gram-positive (*Staphylococcus aureus*) and gram-negative (*Escherichia coli*) bacteria.

## Materials and methods

### Materials

*Launaea cornuta* leaves were collected randomly from the fields of the Nelson Mandela African Institution of Science and technology, Nambala village in Arusha region, Tanzania wat 3° 23′ 58″ S 36° 47′ 48″ E coordinates. The plant was identified and deposited at National Herbarium of Tanzania (NHT) at the Tanzania Plant Health and pesticides Authority (TPHPA) with reference no. KA51/2023. The collection of the plant materials adhered to applicable institutional, national, and international laws and guidelines.

AgNO_3_ (99.99%), hydrous Zn(NO_3_)_2_ (99.99%), NaOH (98%), Ciprofloxacin (98%) and ethanol (99.5%) used in this experiment were purchased from Sigma-Aldrich Chemicals (Germany) and were used without any further purification. The solutions of these analytical precursors were prepared by double distilled water. *Staphylococcus aureus* (ATCC 25923) and *Escherichia coli* (ATCC 25922) bacterial strains were obtained from the laboratory of The Nelson Mandela African Institution of Science and Technology (NM-AIST) Arusha Tanzania.

### Methods

#### Preparation of *Launaea cornuta* leaf extract

The collected *Launaea cornuta* leaves were washed three times with double distilled water to remove sand and any other impurities. The washed leaves were left for 6 h to drain water used in the washing process. 100 g of the *Launaea cornuta* leaves were sliced into small pieces and mixed with 50 mL double distilled water then ground into a fine paste using an electric blender. The resulting paste was introduced into shaking on an electrical shaker for 24 h to allow maximum extraction of the secondary metabolites (phytochemical compounds) from the *Launaea cornuta* leaves. The paste was centrifuged for 20 min under 4000 rpm to separate the leaf extract from the leaf residues. The aqueous extract was further filtered using Whatman No. 1 filter paper to make sure all the leaf particles are removed from the extract. The final obtained *Launaea cornuta* leaf extract with a pH of 5.6 was stored at 4 °C, ready for the green synthesis of Ag-ZnO NCs.

#### Synthesis of Ag, ZnO nanoparticles and Ag-ZnO nanocomposite

##### Biosynthesis of Ag nanoparticles

Ag NPs were synthesized based on the method reported in literature with some modifications [1]. AgNO_3_ was dissolved in double distilled water to prepare a 0. 1 M AgNO_3_ stock solution. 92 mL of *Launaea cornuta* leaf extract was added in a 150 mL beaker under vigorous stirring, 8 mL of 0.1 M AgNO_3_ solution was added dropwise to the leaf extract to make a 8 mM solution. The flask was left to stir for 2 h at a 70 °C, pH was adjusted to 7 using 0.1 M NaOH. The resulting solution was centrifuged and the solid particles obtained were washed twice by 50% ethanol before oven drying for 12 h under 100 °C and then calcined at 650 °C for 3 h. The Ag NPs were stored in an air tight container ready for characterization and antimicrobial test.

##### Biosynthesis of ZnO nanoparticles

Synthesis of ZnO NPs and Ag-ZnO NCs was adopted from Mtavangu et al. [25] with modifications. 92 mL of *Launaea cornuta* leaf extract was placed in a 150 mL beaker under vigorous stirring, 2.9748 g of Zn(NO_3_)_2_.6H_2_O salt was added to the leaf extract under stirring. The reaction was left to continue for 2 h under constant stirring at a 70 °C temperature and pH 7. The resulting solution was centrifuged, and the paste was washed twice by 50% ethanol before oven drying 12 h under 100 °C and then calcined at 650 °C for 3 h to obtain ZnONPs. The ZnONPs were stored in an air tight container ready for characterization and antimicrobial test.

##### Biosynthesis of Ag-ZnO nanocomposite

92 mL of *Launaea cornuta* leaf extract was placed in a 150 mL beaker under vigorous magnetic stirring, 8 mL of 0.1 M AgNO_3_ solution was added dropwise to the leaf extract to make a 8 mM solution. After 20 min 2.97 g of Zn(NO_3_)_2_ salt was added and the pH was adjusted to 7 by using 0.1 M NaOH. The flask was wrapped with aluminum foil and the solution was left to stir for 2 h at a 70 °C temperature. The resulting Ag–ZnO solution was centrifuged, and the paste was washed twice by 50% ethanol before oven drying 12 h under 100 °C and then calcined under 650 °C for 3 h to get Ag–ZnO NCs. The Ag–ZnO NCs were stored in a dry container ready for characterization and antimicrobial test. The reaction was repeated by altering the reaction temperature (30, 50, 70, and 90 °C), different pH (5, 7, 9 and 11, and 13), and different Ag^+^ concentrations (4, 6, 8, and 10 mM) to identify optimum synthesis conditions.

##### Material characterization

The crystalline nature of synthesized nanomaterials was analysed using Rebaku SmartLab X-ray diffractometer (XRD) equipped with analyser scanning mode of CuKα wavelength (λ) = 1.54059 Å, 40 kV–30 mA and 2θ/θ with the spectrum range between 30° and 80°, 0.02 step and 0.2 speed [25]. The identification of functional groups was accomplished through attenuated total reflection-Fourier transform infrared (ATR-FTIR) spectroscopy using the Bruker Optic GmbH 2011 (alpha model, Laser class 1) instrument operating in transmittance mode. Spectral analysis was conducted within the range of 4000–400 cm^−1^ with a spectral resolution of 2 cm^−1^. The formation of silver nanoparticles (Ag NPs) and silver-zinc oxide nanocomposites (Ag–ZnO NCs) was assessed by recording the ultraviolet–visible (UV–Vis) absorption spectra using a UVmini-1240 Shimadzu spectrophotometer from 250 to 800 nm. The particle size, shape, and morphology of the synthesized nanoparticles were characterized by high-resolution transmission electron microscopy—HR-TEM (JEOL JEM 2100, 80–200 kV, Jeol Ltd. Japan) and Field emission scanning electron microscopy (FE-SEM) while the elemental composition was characterized by EDX.

##### Antimicrobial assay

The antimicrobial efficacy of the produced silver nanoparticles (Ag NPs), zinc oxide nanoparticles (ZnO NPs), and silver-zinc oxide nanocomposites (Ag–ZnO NCs) was evaluated against gram-positive (*Staphylococcus aureus*-ATCC 25923) and gram-negative (*Escherichia coli*-ATCC 25922) bacterial strains using the disc diffusion method [15]. Sterilized nutrient agar media was poured into sterilized Petri dishes and allowed to solidify. Upon solidification, individual agar plates were inoculated with each bacterial strain and uniformly spread using a sterilized swab. Absorbent discs, 6 mm in diameter and sterilized, were immersed in colloidal solutions of Ag NPs, ZnO NPs, and Ag–ZnO NCs with concentrations ranging from 10 to 50 mg/mL. These soaked discs were then placed on the inoculated Petri dishes. After 24 h of incubation at 37 °C, the bacterial inhibition results were interpreted and presented in terms of the zone of inhibition (ZOI) and minimum inhibition concentration (MIC) in millimeters. Ciprofloxacin served as the positive control in this study. Additionally, the antimicrobial activity of *Launaea cornuta* leaf extract (LE) was assessed since it is traditionally used in medicine.

## Results and discussion

### X-ray diffraction

**Fig. 1:**
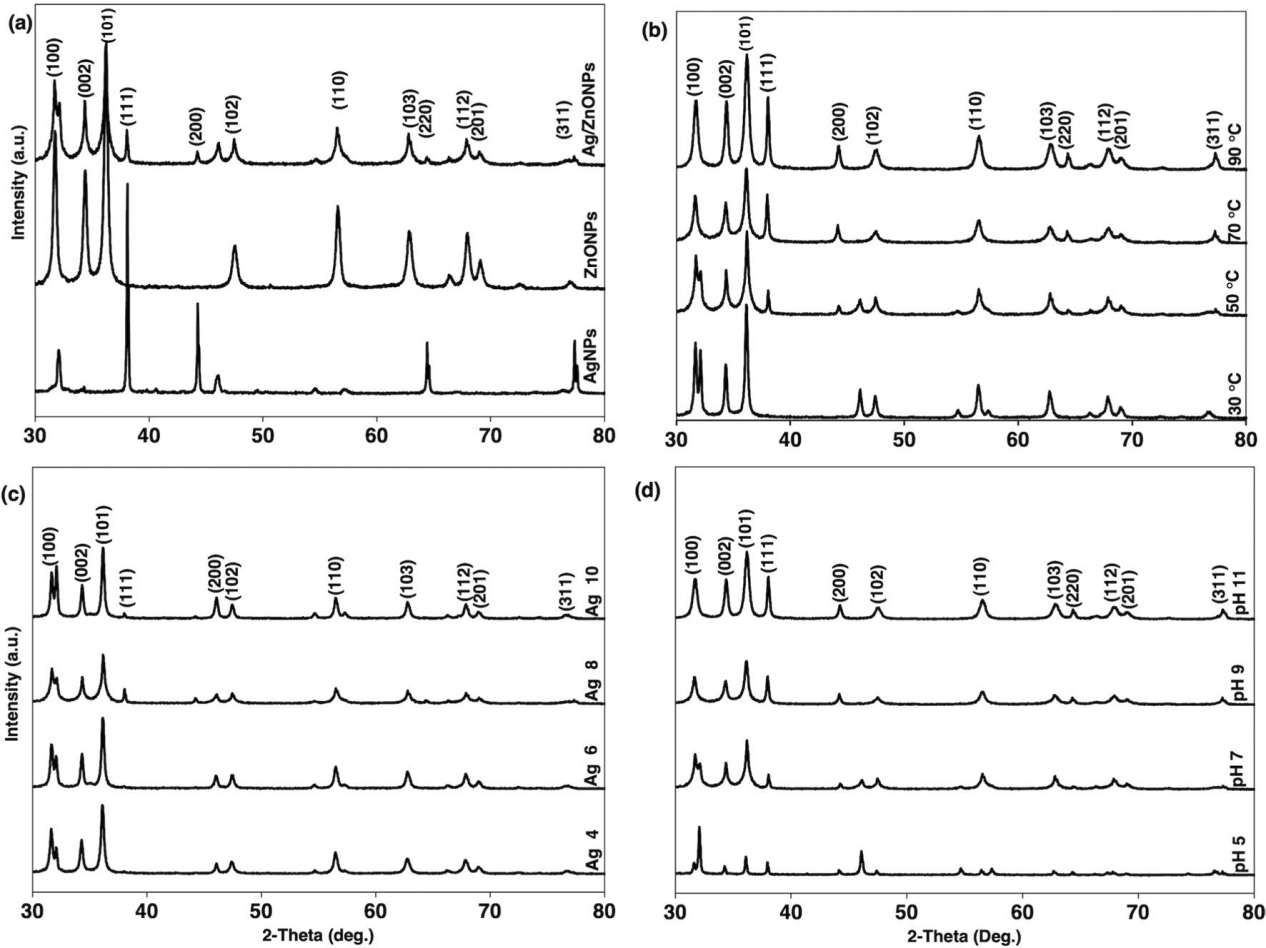
XRD patterns of **a** pure Ag NPs, ZnO NPs and Ag–ZnO NCs, **b** Ag–ZnO NCs at different temperature, **c** Ag–ZnO NCs at different Ag^+^ concentration and **d** Ag–ZnO NCs at different pH

Figure 1 diffractogram shows the XRD patterns of pure Ag NPs, ZnO NPs, and the Ag–ZnO NCs. It shows the Ag–ZnO NCs patterns with different Ag^+^ content, synthesis temperature, and pH. The pure Ag NPs pattern indicated in Fig. 1a shows four strong peaks at 2θ values of 38.10, 44.28, 64.44, and 77.38 degrees which correspond to the crystal planes of (111), (200), (220), and (311). These patterns confirm the face-centered-cubic (fcc) crystalline nature of the synthesized AgNPs with lattice parameters a = b = c = 4.088 A° as compared to the silver standard powder diffraction card, JCPDS, file No. 04-0783 [59, 60]. The pure ZnO XRD patterns display eight diffraction peaks at 2θ values of 31.74°, 34.38°, 36.18°, 47.42°, 56.48°, 62.80°, 67.84°, and 68.24° corresponding to the crystal planes (100), (002), (101), (102), (110), (103), (112), and (201). The crystal planes matched well with the ZnO standard powder diffraction card JCPDS, File No. 01-076-0704 indicating the formation of the typical hexagonal wurtzite structure space group P 63 mc ZnO NPs in the matrix [24, 61]. Furthermore, the XRD pattern of the Ag–ZnO NCs exhibits the peaks that correspond to both Ag and ZnO, proof that Ag–ZnO NCs have been synthesized. These patterns are indications of peaks appeared at 2θ values of 31.74°, 34.38°, 36.18°, 38.10°, 44.28°, 47.42°, 56.48°, 62.80°, 64.44°, 67.84°, 68.24° and 77.38° corresponding to the crystal planes (100), (002), (101), (111), (200), (102), (110), (103), (220), (112), (201) and (311). The intensity of the Ag NPs peaks decreased in the composite as a result of successful doping by ZnO forming a core around the Ag NPs [62]. Figure 1b shows the effect of temperature in the synthesized Ag–ZnO NCs especially on the peaks representing the presence Ag NPs in the composite. The increase in synthesis temperature has led to an increase in intensity of peaks: (111), (200), (220), and (311). This suggests the formation of Ag NPs at high temperatures. This is attributed to the increased collision of the reacting species leading to the formation of more nuclei [41]. Figure 1c shows the effect of the reaction materials concentration. In this study, the concentration of the Ag^+^ used was varied to evaluate its effect. It has been observed that as the Ag concentration increases the peak intensity increases. At low concentrations, the Ag NPs peaks were almost invisible. It might be contributed to being overshadowed by the ZnO NPs which surrounded the Ag NPs. Figure 1d shows the increased intensity of the composite peaks as the pH of the reacting solution changed from acidic to alkaline. This provides insight into the formation of more crystalline particles at around neutral to alkaline conditions during the synthesis process.

For the precise calculation of the average particle size of the synthesized materials the Debye–Scherrer equation represented by Eq. (1) was used where, K is the Scherrer constant (0.94), λ is the specific wavelength of the X-ray used which is 0.154 nm, β is the full width at half maxima, θ is the diffraction Bragg angle and D is the average crystallite size in nm.

1 $$D = \frac{{{\text{\rm K}}\lambda }}{{\beta_{2\theta } \cos \theta }}$$

From this equation, the average crystallite size for pure Ag NPs, ZnO NPs, and Ag–ZnO NCs were 50.6, 22.1 and 21.5 nm respectively. Ag doping on ZnO has caused a subsequent decrease in the average crystal size of Ag which might be attributed to the dispersion of ZnO nanoparticles around the Ag lattice, which limits the coalition of Ag NPs, thus hindering its agglomeration. The average particle size decreased as the temperature increased, with values of 35.1 nm at 30 °C, 30.6 nm at 50 °C, and 21.5 nm at 70 °C. The increase in the reaction temperature leads to an increase in the reduction rate of the Zn^2+^ and Ag^+^ ions. It also increases the subsequent homogeneous nucleation of ions causing the formation of small-size Ag–ZnO NCs [41, 63]. The particle size for the composites synthesized at pH 5, 7, 9, and 11 was 43.3, 21.5, 22.1 and 24.3 indicating the smallest particles to be obtained at the neutral pH [42, 64]. The Ag–ZnO NCs synthesized at 4, 6, 8, and 10% Ag had the average particle sizes 29.3, 26.8, 21.5 and 28.8 nm, respectively. Similar results reported were elsewhere [25].

### Optical properties

#### ART-FTIR studies

**Fig. 2:**
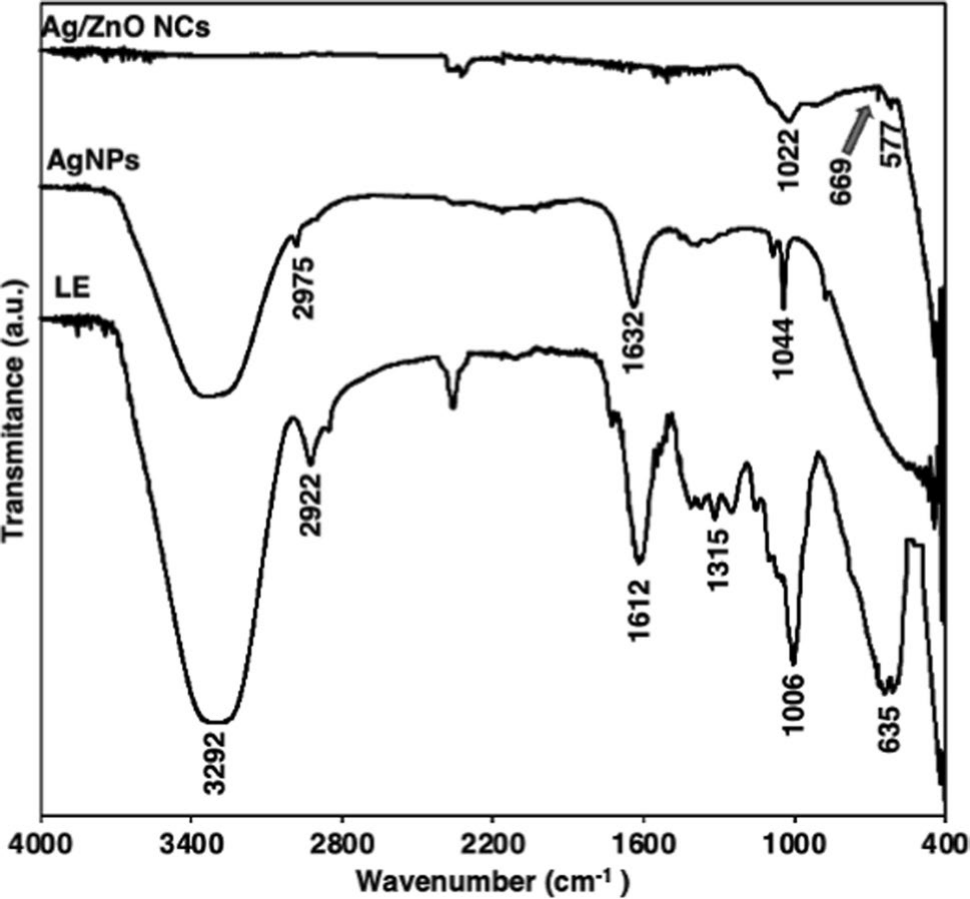
FTIR patterns of leaf extract, Ag NPs and Ag–ZnO NCs

FTIR spectrum of the *Launaea cornuta* leaf extract indicates the presence of alkaloids, saponins, flavonoids, and tannins as secondary metabolites responsible for reducing and capping of metal ions and nanoparticles respectively. Figure 2 indicates that FTIR spectra of *Launaea cornuta* leaf extract exhibited the peaks at a wavenumber 635 cm^−1^ which is attributed to the N–H stretching vibrations. The 1315 and 1006 cm^−1^ are attributed to the C–N stretching vibrations of the aromatic and aliphatic amines, respectively [65]. The presence of N–H and C–N peaks signifies the presence of aliphatic amine groups which confirms the presence of alkaloids [66, 67] which act as hydrolyzing/reducing agents in the formation of nanoparticles and nanocomposites. The strong and broad band at 3292 cm^−1^ indicates O–H and N–H stretching vibration [68] while the band at 1315 cm^−1^ indicates C–C starching [67]. These bands indicate the presence of flavonoids and tannins [51, 69]. The 1612 and 2922 cm^−1^ bands correspond to the C=C and C–H vibrations which indicates the presence of saponins in the leaf extract. The saponins, flavonoids, and tannins act as natural surfactants and capping agents controlling particle size growth and preventing agglomeration by providing a steric hindrance on the nanoparticles formed [70]. The shortening and disappearance of the O–H vibration observed after the biosynthesized Ag and Ag–ZnO NCs respectively may be due to the binding of the -OH on the surface of the nanoparticles and volatilization during calcination [51, 71]. The FTIR of the synthesized Ag NPs indicates a shortening of the 3292 cm^−1^ band, disappearing of 1315 and 635 cm^−1^ band as well as a shift of other bands from 2922 to 2975 cm^−1^, 1612 to 1632 cm^−1^, and 1006 to 1044 cm^−1^. In the case of Ag–ZnO NCs there is a disappearance of the 3292, 2922, 1315, and 1612 cm^−1^ bands with a shift from 1006 to 1022 cm^−1^ and 635 to 577 cm^−1^ bands. These changes suggest the binding of the Ag and ZnO on the proteins through the free amine groups, hydroxyl and carboxylate ions of amino acid [41, 72]. The 6 cm^−1^ stretching peak indicates the presence of the synthesized ZnO in the biosynthesis process [73, 74].

#### UV–Vis spectrum

**Fig. 3:**
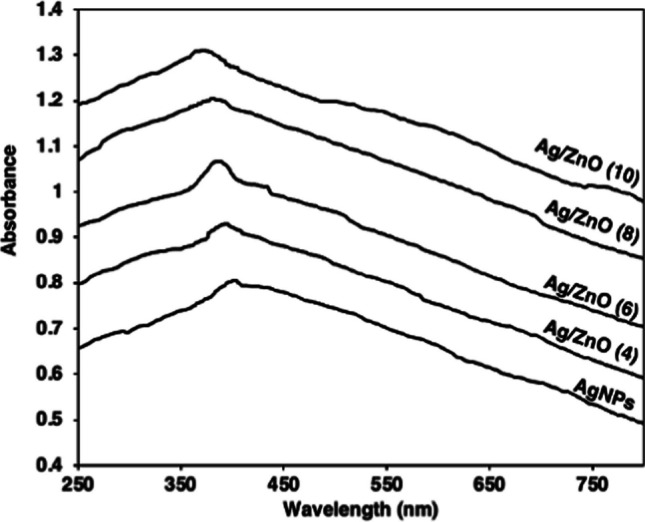
UV–Vis patterns of Ag NPs and Ag–ZnO NCs in 4, 6, 8, and 10% Ag^+^

The aqueous leaf extract of *Launaea cornuta* was used for the green synthesis in this study. During the synthesis process the leaf extract color changed from light brown to brown after 10 min of addition of AgNO_3_ solution becoming dark brown on addition of Zn(NO_3_)_2_ This is an initial indication of the formation of Ag NPs and ZnO NPs in the solution [75, 76]. The color change is a result of the excitation of the surface plasmon vibrations in the Ag NPs which causes color change in the solution [12, 14, 71, 75]. The UV–Vis spectrometry as illustrated in Fig. 3 depicts the absorption band peak at 403 nm providing evidence of the formation of Ag NPs [47, 77]. The addition of Zn(NO_3_)_2_ to form Ag–ZnO NCs, shifted the absorption band peaks to around 372 and 394 nm as the concentration of silver increased from 4 to 10%. This is the evidence of the presence of ZnO on the prepared nanomaterials [17, 78]. In the investigation of the effect of doping concentration, 4, 6, 8, and 10% Ag^+^ were applied. In these concentrations, adsorption peaks were observed at 394, 386, 381, 372 nm. The increase of AgNO_3_ concentration resulted in the shift of the peaks from 403 to 372 nm which is referred to as blue shift. The blue shift is a result of the Ag NPs attachment around ZnO NPs leading to the formation of smaller nuclei on the ZnO surface therefore decreasing the nanoparticles size [25, 79].

### Morphological studies

#### Field emission scanning electron microscopy (FE-SEM)

**Fig. 4:**
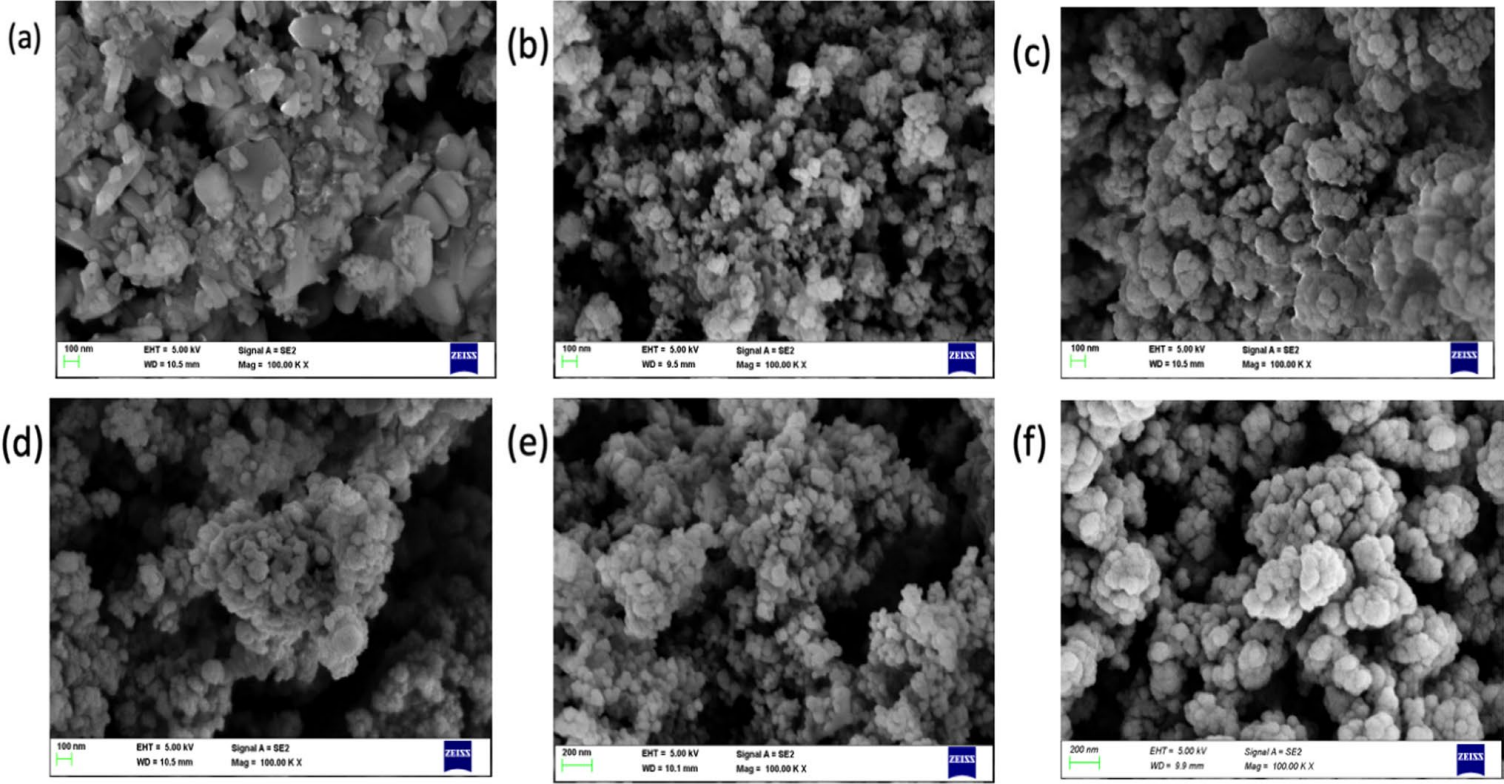
SEM images of **a** pure Ag NPs, **b** pure ZnO NPs, and Ag–ZnO NCs with **c** 4%, **d** 6%, **e** 8% and **f** 10% Ag concentrations

**Fig. 5:**
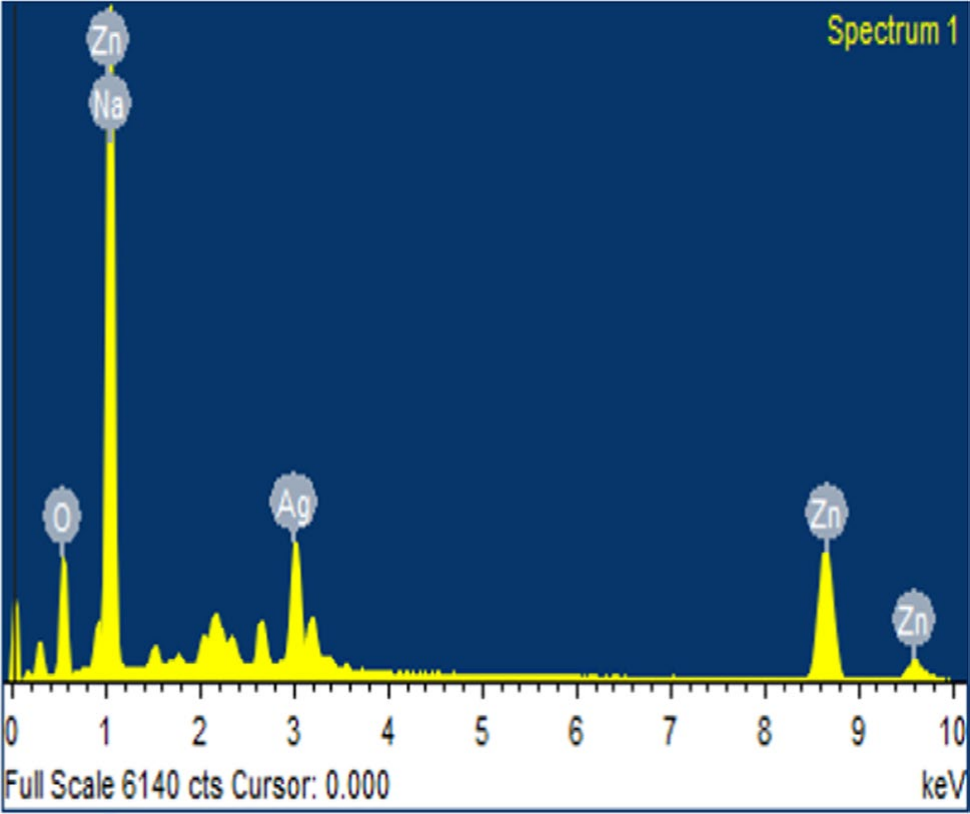
EDS spectra of Ag–ZnO NCs

Figure 4 illustrate the morphology of the synthesized nanomaterials by using *Launaea cornuta* leaf extract at different AgNO_3_ concentrations. The FE-SEM images show that AgNO_3_ concentration had significant effects on the morphologies of the synthesized nanoparticles [41, 42, 63, 64]. Ag NPs indicated in Fig. 4a were observed to have spherical, tubelike, and blocklike aggregated nanoparticles with an average particle size of 75.7 nm [63]. ZnO NPs in Fig. 4b displayed spherical agglomerated particles with an average particle size of 33.09 nm. Doping of the Ag NPs into ZnO had an effect on the shape and size of the resulting Ag–ZnO NCs composite. Ag-ZnO NCs demonstrated spherical agglomerated composites with the average particle size average of 36.11 nm. The decrease of particle size from pure Ag NPs to Ag–ZnO NCs might be attributed to the Ag NPs anchoring on the surface of ZnO NPs [80]. The average particle size for the composites synthesized at 30, 50, 70, and 90 °C were 46.75, 43.5, 36.11, and 39.29 nm respectively. As the temperature increased the particle size decreased, and the results correspond to the XRD results discussed above. The FE-SEM images, Fig. 4c–f, of the composites synthesized at concentrations 4, 6, 8, and 10% Ag^+^ demonstrated particle size averages of 51.54, 50.16, 46.11, and 49 nm respectively decreasing as the Ag^+^ percentage increases, a trend proved by the XRD results of this study. The smallest particle size was obtained at pH 7 condition. As the pH increases there is an increase in particle size caused by the increased nucleation that supports hydrolysis and accelerates the particle size growth due to agglomeration [81]. EDS spectra in Fig. 5 further proves the presence of Ag, Zn and O in the Ag–ZnO NCs synthesized samples. It is a proof that *Launaea cornuta* leaf extract can be used successfully to synthesiwe Ag–ZnO NCs.

#### High-resolution transmission electron microscopy (HR-TEM)

**Fig. 6:**
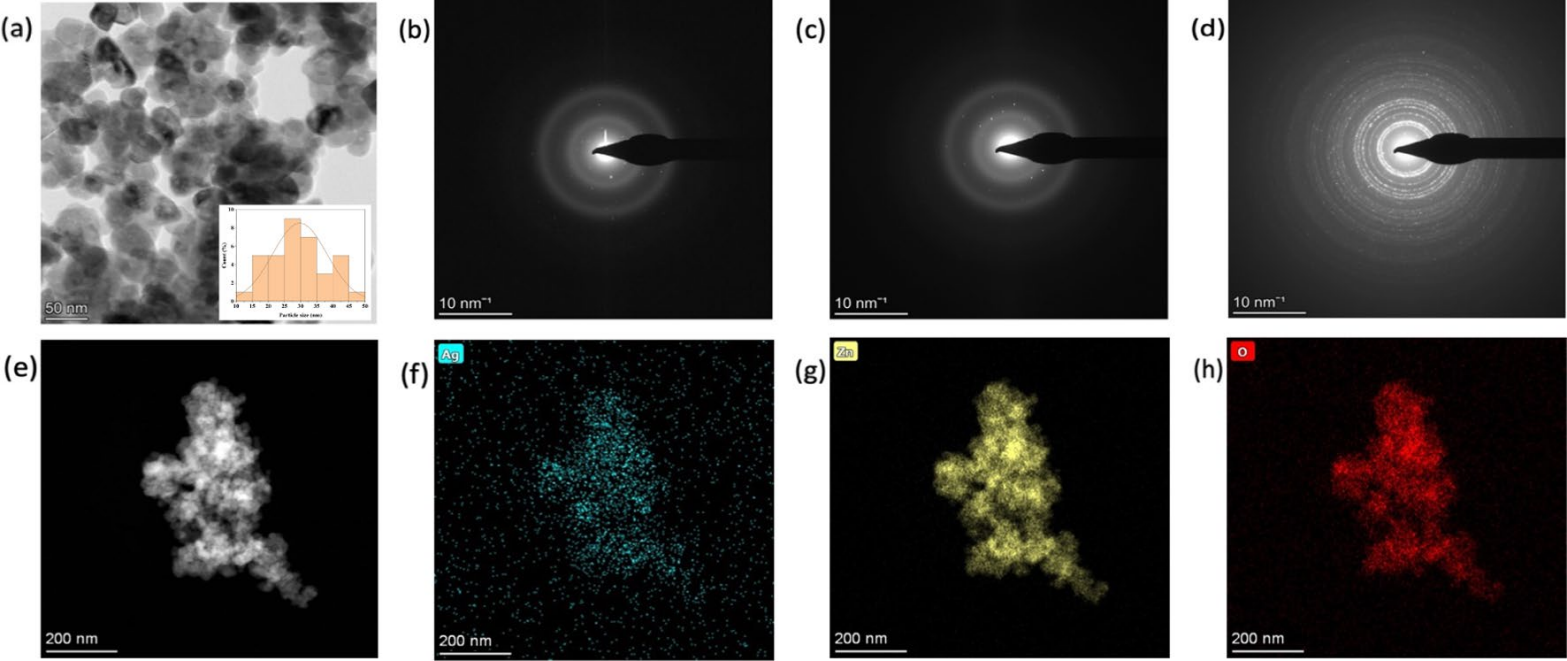
**a** TEM of the Ag–ZnO NCs (the inset is particle distribution histogram), SAED patterns of **b** Ag NPs, **c** ZnO NPs, **d** Ag–ZnO NCs, and the EDAX mapping of **e** Ag–ZnO, **f** Ag, **g** Zn, and **h** O on the Ag–ZnO NCs

Figure 6a is the TEM of the Ag–ZnO NPs showing a dispersion of the spherical ZnO nanoparticles anchored on the surface of Ag nanoparticles which resulted to the controlled agglomeration of Ag NPs therefore decreased particle size. The particle size distribution of Ag–ZnO NCs ranges from 13.89 to 45.37 nm as illustrated in the histogram (inset in Fig. 6a) and its average particle size was 29.75 nm. The average particle size of Ag NPs and ZnO NPs were 59.78 and 26.5 nm respectively which corresponds to the result patterns of XRD and SEM. The addition of Ag NPs in the ZnO NPs matrix decreases the average particle size of Ag NPs by more than 50%. The selected area electron diffraction (SAED) pattern of Ag NPs, ZnO NPs and Ag–ZnO NCs indicate concentric rings as shown in Fig. 6b–d. The organized circular pattens explains the crystalline nature of prepared nanomaterials. The SAED pattern of Ag–ZnO NCs shows the increase of the cycles indicating the presence of both Ag and ZnO in the composite. The EDAX mapping of Ag–ZnO NCs indicated the composition and distribution of atoms present in the composite (Fig. 6e–h). The EDAX mapping demonstrates a consistent dispersion of Ag, Zn, and O within the composite material, confirming the successful incorporation of Ag into ZnO, as illustrated in Fig. 6e, f. These findings provide strong evidence that silver ions have been effectively integrated into the ZnO nanoparticles.

### Antimicrobial assessment

**Fig. 7:**
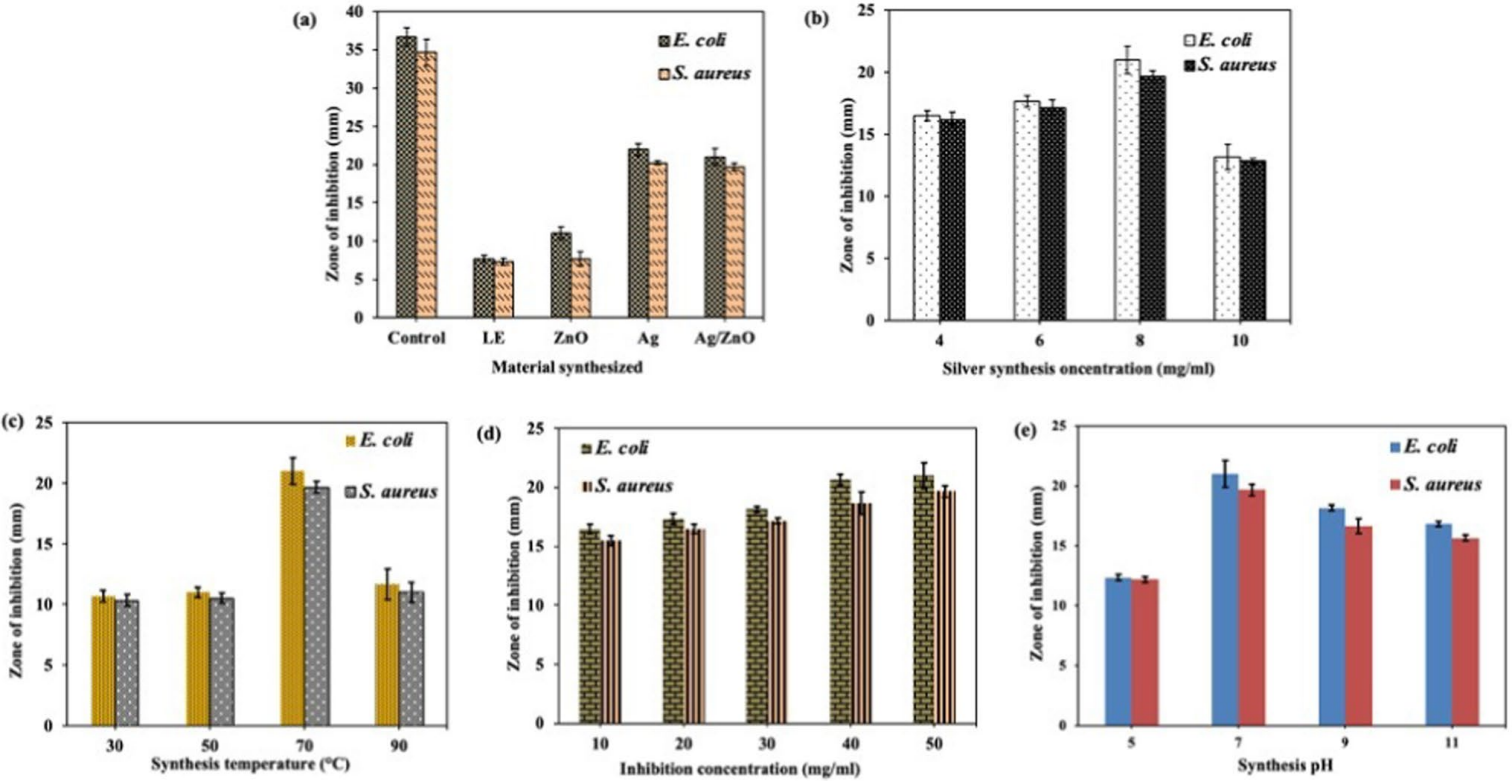
Zone of inhibition for the synthesized nanoparticles on *E. coli* and *S. aureus* **a** pure Ag NPs, ZnO NPs, Ag–ZnO NCs, leaf extract and control **b** Ag–ZnO NCs at different Ag^+^ synthesis concentration, **c** Ag–ZnO NPs at different synthesis temperature **d** Ag–ZnO NCs at different inhibitory concentration and **e** Ag–ZnO NCs at different pH

Studies report a remarkable reduction in microbial growth when exposed to Ag NPs, ZnO NPs, and Ag–ZnO NCs [13, 77, 82–84]. In this study, antimicrobial activity of Ag NPs, ZnO NPs, and Ag/ZnO NCs against gram-negative (*E. coli*) and gram- positive (*S. aureus*) bacteria strains were examined. The effect of synthesis concentration, temperature, and pH were investigated. Figure 7a indicates the ZOI for ciprofloxacin indicated as “control”, *Launaea cornuta* leaf extract (LE) and the synthesized Ag NPs, ZnO NPs, and Ag-ZnO NCs. The existence of the zone of inhibition around the NPs indicates that the NPs are antimicrobial active. Ciprofloxacin exhibited an inhibition zone of 36.7 ± 1.2 and 34.7 ± 1.7 mm for *E. coli* and *S. aureus* respectively. The *Launaea cornuta* leaf extract (LE) was found to inhibit bacteria growth with the ZOI of 7.7 ± 0.5 and 7.3 ± 0.5 mm for *E. coli* and *S. aureus* respectively. As indicated in Fig. 7a Ag NPs have demonstrated the highest ZOI for both bacterial strains among the synthesized NPs followed by Ag–ZnO NCs and then ZnO NPs, as reported elsewhere [9, 85]. The highest ZOI of Ag NPs was 22.0 ± 0.8 mm and 20.2 ± 0.2 mm, for *E. coli* and *S. aureus* while that of Ag-ZnO NCs was 21 ± 1.1 mm and 19.7 ± 0.5 mm respectively. ZnO NPs had a ZOI of 11.0 ± 0.8 mm and 7.7 ± 0.9 mm, for *E. coli* and *S. aureus.* The *p* values for both strains were < 0.05 proving the significance of the results obtained. In this study, the *E. coli* (gram-negative) inhibition was higher than *S. aureus* (gram-positive) bacteria. This perspective is contributed by the properties of the cell wall and membrane of the bacteria and the nature or properties of the NPs. Gram-negative bacteria have a thin peptidoglycan layer, compared to gram-positive bacteria which has thick and rigid peptidoglycan layer crosslinked by peptide chains. The thin layer of gram-negative bacteria facilitates easy penetration of the NPs to the bacterial nuclei, therefore, limiting cell regeneration [25, 86]. On the other hand, there is an electrostatic interaction between the positive NPs and the gram-negative bacterial membrane which is negatively charged. The attraction facilitates easy penetration of the NPs into the bacterial cell unlike gram-positive bacterial cells [87, 88]. The Ag–ZnO NCs were synthesized in four different temperatures, 30, 50, 70, and 90 °C to establish optimum conditions. According to the inhibition results, the highest inhibition was demonstrated by the NPs synthesized at 70 °C as indicated in Fig. 7c. Ag–ZnO NCs obtained above 70 °C had low microbial inhibition which might be attributed to the increased nucleation rate that leads to large particle size formation [36, 50]. The effect of silver doping concentration was also studied, and the increase in concentration increased the bacterial growth inhibition as demonstrated in Fig. 7b. This signifies that silver is a good antimicrobial agent [89, 90]. Figure 7d shows a positive correlation between the increased inhibition concentration from 10 to 50 mg and increased microbial inhibition for both strains as a result of increased dosage concentration of the nanoparticles per bacterial strain. The synthesis pH for Ag–ZnO NCs was also investigated and found to give the best inhibition results at pH 7 as indicated in Fig. 7e. These results correspond to the XRD results discussed above which shows at this pH the NPs have the smallest average size.

In this research, green synthesized nanomaterials have displayed exceptional effectiveness in inhibiting the growth of both gram-negative and gram-positive bacteria, surpassing previously reported research outcomes. These findings, highlighted in Table 1, reveal significantly lower minimum inhibition concentrations of the green synthesized nanoparticles, underscoring their potential to revolutionize antimicrobial strategies. This research not only showcases the promise of eco-friendly synthesis methods but also underscores their fundamental role in the development of highly potent antibacterial agents.

**Table 1:** Comparison of antimicrobial activity of Ag NPs, ZnO NPs and Ag–ZnO NCs of this study with other studies

Plant extract	Bacteria	Nanoparticles	Crystollite size (nm)	Concentration (MIC)	Zone of inhibition (mm)	References
*Ocimum tenuiflorum* seed extract	*Escherichia coli*	Ag–ZnO	54	2 mg/mL	Total inhibition in 15 min	[6]
*Trigonella foenum-graecum* leaf extract	*Staphylococcus aureus*	Ag–ZnO		20 mg/mL	13.5 ± 0.707	[91]
	*Escherichia coli*	Ag–ZnO		20 mg/mL	12.5 ± 0.707	[91]
*Suaeda aegyptiaca* leaf extract	*Staphylococcus aureus*	ZnO	60	10 mg/mL	16.01	[92]
*Impatiens balsamina*	*Staphylococcus aureus*	Ag	20.33	–	13.8	[1]
	*Escherichia coli*	Ag	20.33	–	8.9	[1]
*Carica Papaya* fruit	*Staphylococcus aureus*	Ag	75.68	169.9 ppm	11	[93]
	*Escherichia coli*	Ag	75.68	169.9 ppm	2	[93]
*Launaea cornuta* leaf extract	*Escherichia coli*	Ag	50.64	10 mg/ml	19.3 ± 0.94	This study
	*Escherichia coli*	ZnO	22.08		9.3 ± 0.83	
	*Escherichia coli*	Ag–ZnO	21.51		16.5 ± 0.46	
	*Staphylococcus aureus*	Ag	50.64		17.67 ± 0.96	
	*Staphylococcus aureus*	ZnO	22.08		6.5 ± 0.41	
	*Staphylococcus aureus*	Ag–ZnO	21.51		15.5 ± 0.56	
Aloe Socotrina leaf extract	*Escherichia coli*	ZnO	15–50	100 µg/mL	25 ± 1.7	[94]
*Pistacia atlantica* resin	*Staphylococcus aureus*	Ag–ZnO	18.9	0.0001 mg/mL	12 ± 0.06	[15]
	*Escherichia coli*	Ag–ZnO	18.9	0.0001 mg/mL	15.2 ± 0.007	[15]

### Antimicrobial mechanism

The antimicrobial mechanism of Ag–ZnO involves the synergistic action of silver (Ag) and zinc oxide (ZnO) nanoparticles. Ag possesses potent antimicrobial properties due to its ability to release silver ions (Ag^+^) that can disrupt bacterial cell membranes and inhibit essential cellular functions resulting in the cellular death of affected bacteria [95, 96]. The Ag^+^ binds to the thiol groups of proteins and enzymes, leading to the inhibition of enzyme activity and DNA replication. The formation of free Ag radical attacks the bacterial membrane lipids causing their dissociation, and damage, and finally inhibits the growth of the bacteria [97]. Additionally, Ag^+^ can be internalized and generate reactive oxygen species (ROS), creating oxidative stress that alters the cell cycle and stimulates cell death through apoptosis or autophagy [77]. Furthermore, it is reported that Ag^+^ binds to the sulfhydryl groups of the metabolic enzymes of the bacterial electron transport chain bringing about their inactivation [98]. On the other hand, ZnO nanoparticles exhibit antimicrobial activity by releasing Zn^2+^ ions that disrupt the integrity of bacterial membranes and interfere with intracellular processes [36, 91]. ZnO nanoparticles can also generate ROS, leading to oxidative stress and cellular damage. The combination of Ag and ZnO nanoparticles in Ag–ZnO nanocomposites enhances the antimicrobial activity by providing a dual mechanism of action, resulting in effective inhibition of various pathogens, including bacteria and fungi.

## Conclusions

Ag–ZnO NCs were successfully synthesized using an environmentally friendly and efficient method by using *Launaea cornuta* leaf extract. The presence of phytochemical compounds in the leaf extract played a crucial role as reducing and capping agents during the synthesis process. The XRD, TEM, and SEM analyses confirmed the spherical and crystalline nature of the composites, with an average particle size of 21.5 nm under optimum conditions. The size and controlled agglomeration of the synthesized nanoparticles were influenced by the synthesis pH, temperature, and silver concentration. The antimicrobial efficacy of the Ag–ZnO NCs was assessed against *E. coli* and *S. aureus* bacterial strains, demonstrating significant inhibitory effects. Notably, the inhibitory activity was more pronounced against *E. coli* (gram-negative bacteria) compared to *S. aureus* (gram-positive bacteria). Thus, the *Launaea cornuta* leaf extract holds great promise as a potential source of phytochemical compounds for the synthesis of Ag–ZnO NCs, replacing synthetic chemicals. Furthermore, the incorporation of ZnO into Ag NPs has proven effective in controlling agglomeration, shape, and particle size, offering a sustainable approach to nanoparticle synthesis.

### Supplementary Information

Additional file1 (DOCX 94 kb)

## Data Availability

The datasets generated and/or analyzed in this study are available in the DRYAD repository and can be accessed through the following link: https://datadryad.org/stash/share/8_UpVqAbW6GsvrRsAznCeEYg65fu1iXAEwXJi-Zrtlw
