# Supplementary material for: Facile biosynthesis of Ag–ZnO nanocomposites using Launaea cornuta leaf extract and their antimicrobial activity
Source: Discov Nano. 2023 Nov 17;18(1):142. doi: 10.1186/s11671-023-03925-2 (PMC10656379; doi:10.1186/s11671-023-03925-2)
Supplement: Supplementary file 1 — Additional file1 (DOCX 94 kb) [file 11671_2023_3925_MOESM1_ESM.docx]

**Facile Biosynthesis of Ag-ZnO Nanocomposites using *Launaea cornuta* leaf extract and their antimicrobial activity**

Elizabeth Makauki^1^*, Stanslaus George. Mtavangu^2,4^, Onita D. Basu^3^, Mwemezi Rwiza^1^, Revocatus Machunda^1^

^1^School of Materials Energy Water and Environmental Sciences, Nelson Mandela African Institution of Science and Technology, Arusha, Tanzania.

^2^Department of Chemical Engineering, Faculty of Engineering Sciences, KU Leuven, Belgium.

^3^Faculty of Graduate & Postdoctoral Affairs, Carleton University, Ottawa, Canada.

^4^Department of Chemistry, Dar es Salaam University College of Education, Dar es Salaam, Tanzania.

**Corresponding email: [elizabeth.makauki@nm-aist.ac.tz](mailto:elizabeth.makauki@nm-aist.ac.tz)*

**SUPPORTING INFORMATION**


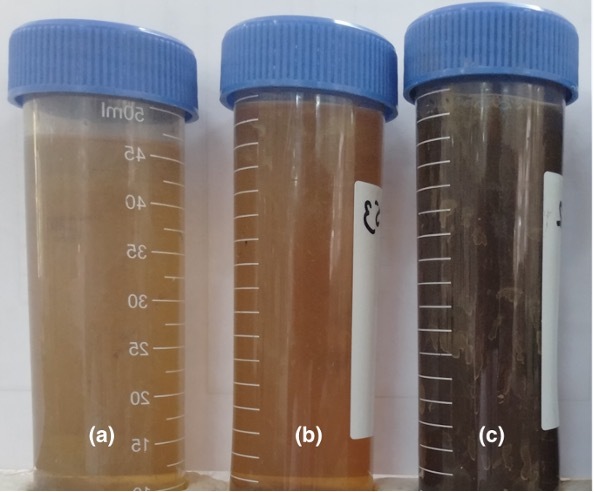


**Fig. 1** Color change during the synthesis of Ag-ZnO NCs: **(a)** *Launaea cornuta* leaf extract, **(b)** after addition of 8 mM AgNO_3_, **(c)** after addition of Zn(NO_3_)_2_ in reaction


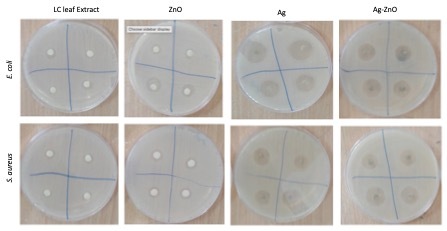


**Fig. 2 ﻿**Real images showing the antibacterial activity of *Launaea cornuta* leaf extract, biosynthesized pure Ag, ZnO and Ag–ZnO nanocomposites on Escherichia coli and Staphylococcus aureus bacterial strains
